# The impact of the COVID-19 pandemic on perinatal loss among Italian couples: A mixed-method study

**DOI:** 10.3389/fpsyg.2022.929350

**Published:** 2022-09-12

**Authors:** Ines Testoni, Lucia Ronconi, Erika Iacona, Alice Trainini, Nella Tralli, Luisella Nodari, Giulia Limongelli, Loredana Cena

**Affiliations:** ^1^Department of Philosophy, Sociology, Education and Applied Psychology (FISPPA), University of Padova, Padua, Italy; ^2^Faculty of Social Welfare and Health Sciences, Emili Sagol Creative Arts Therapies Research Center, University of Haifa, Haifa, Israel; ^3^IT and Statistical Services, Multifunctional Centre of Psychology, University of Padova, Padua, Italy; ^4^Perinatal Clinical Psychology Observatory, Department of Clinical and Experimental Sciences, University of Brescia, Brescia, Italy

**Keywords:** perinatal grief, COVID-19, pandemic, mixed-method research design, psychological support

## Abstract

**Background:**

Perinatal bereavement is an event that greatly impacts the emotional, psychological, and psychosocial aspects of those who want to have a child.

**Objectives:**

Since there are few studies on the psychological impact of the COVID-19 pandemic on couples grieving for perinatal loss, this research aimed to survey this experience.

**Participants:**

Between 2020 and 2021, in Italian provinces highly affected by the COVID-19 pandemic, 21 parents participated: 16 mothers (76%; mean age 36.2; SD: 3.1) and 5 fathers (24%; mean age 40.2; SD: 3.4), among which there were 4 couples.

**Methods:**

A mixed-method design was used through self-report questionnaires and in-depth interviews. Accompanied by a sociodemographic form, the following questionnaires were administered: Prolonged Grief-13, the Parental Assessment of Paternal Affectivity (PAPA) (to fathers), the Parental Assessment of Maternal Affectivity (PAMA) (to mothers), the Dyadic Adjustment Scale short version, the Daily Spiritual Experiences Scale, and the Impact of Event Scale-Revised. The texts obtained through the in-depth interviews underwent thematic analysis.

**Results:**

Fifty per cent of participants suffered from Post-Traumatic Stress Disorders (PTSD) symptoms and 20% suffered from relational dyadic stress. Four areas of thematic prevalence emerged: psychological complexity of bereavement, the impact of the COVID-19, disenfranchisement vs. support, and spirituality and contact with the lost child. Participants interpreted their distress as related to inadequate access to healthcare services, and perceiving the pandemic restrictions to be responsible for less support and lower quality of care. Furthermore, they needed psychological help, and most of them were unable to access this service. Spirituality/religiosity did not help, while contact with the fetus and burial did.

**Conclusion:**

It is important to implement psychological services in obstetrics departments to offer adequate support, even in pandemic situations.

## Introduction

Perinatal loss, comprised of experiences of miscarriage, stillbirth, and neonatal death, is a highly traumatic experience characterized by stressful conditions aggravated by the psychological dissolution of expectations regarding raising a child and developing a parental role (Obst et al., 2020; Testoni et al., 2020). Experiences of loss are similar at every stage of gestation up to the first month of the child’s life (Callister, 2006) and have a deeply negative impact on mothers’ psychological and physical wellbeing (Kersting and Wagner, 2012). Typical feelings are sadness and despair, anxiety, guilt, anger, and longing for the child, which are accompanied by physiological changes, such as sleep disturbances and lack of appetite, and psychiatric symptoms, including complicated grief (Kersting et al., 2011). Guilt mainly occurs, especially in the presence of ambivalent feelings toward the pregnancy (Kersting and Wagner, 2022), the conviction of having made mistakes, and the perception of failure of the body (Jones, 2014). This condition may persist for several months or even years (DeMontigny et al., 2017), and the couple’s relationship is often undermined because of feelings of guilt, mutual attribution of responsibility, resentment, and the perceived failure of the parental project (Kersting and Wagner, 2012; Cena and Stefana, 2020).

The suffering of loss may be exacerbated and prolonged due to lack of social or partner support (Lasker and Toedter, 2000; Burden et al., 2016), an unsatisfactory relationship with healthcare staff (DeMontigny et al., 2017), lack of funerals or other rituals (Kersting and Wagner, 2012), and perceived social delegitimization of grief (Capitulo, 2005). On the contrary, protective factors are social support (Toedter et al., 2001), membership in religious communities (McIntosh et al., 1993), having children, and a satisfactory relationship with healthcare professionals (DeMontigny et al., 2017; Cena et al., 2021a). The literature on the effects of COVID-19 on maternity is contradictory, as is the information from the media. It seemed that pregnant women affected by infectious diseases did not appear to report more severe symptoms than non-pregnant women and, in most cases, were asymptomatic or mildly symptomatic (Delahoy et al., 2020). However, studies have also shown that adverse outcomes following maternal COVID-19 infection were infrequent (Delahoy et al., 2020), and others have evidenced that pregnant women were more likely to be intubated and develop composite morbidity (DeBolt et al., 2021; Kotlar et al., 2021) or die (Zambrano et al., 2020). The risk of COVID-19 transmission from mother to fetus is very low (Egloff et al., 2020); however, some studies point to an increase in premature births (Blitz et al., 2020; Lokken et al., 2020; Allotey et al., 2021), low birth weight, cesarean sections (Knight et al., 2020; Savasi et al., 2020), and maternal and neonatal mortality, and extrauterine pregnancies compared with the pre-pandemic period (Khalil et al., 2020; Chmielewska et al., 2021). As infodemia had a negative effect on pregnant women (Ennab et al., 2022), it is possible to hypothesize that some concerns inherent in COVID-19 could have impacted pregnant women and their partners who lost their babies. Since the literature is still scarce on this issue, this study wanted to consider this particular group of persons.

## The research

### Objectives and participants

The aims of the research were to investigate the experiences related to the impact of the COVID-19 pandemic on mothers and fathers’ grief who experienced a perinatal loss between 2020 and 2021. Changes in social and couple relationships, maternal/paternal affectivity and satisfaction, trauma, grieving strategies, and un/helpful factors were detected.

They were recruited at healthcare centers throughout Italy (counseling centers, hospitals, etc.) by healthcare professionals (psychologists, psychotherapists, psychiatrists, midwives etc.), collaborating with the Observatory of Perinatal Clinical Psychology at University of Brescia.

Psychotherapists collaborating with family counseling centers identified potential participants and indicated them to the researchers of this study. The inclusion criteria were as follows: the loss occurred from March 2020 to March 2021; being able to speak the Italian language. Mental health was the main exclusion criterion: participants were not undergoing psychiatric or psychopharmacological treatment. Recruitment of participants ended when the topics brought in by the research participants became repetitive and the data reached theoretical saturation (Morse, 2015).

Twenty-one parents participated: 16 mothers (76%; mean age 36.2; SD: 3.1) and 5 fathers (24%; mean age 40.2; SD: 3.4). Among them, 86% had Italian nationality; 62% had a university degree, 33% had a high school diploma and 5% had a middle school license; 95% is employed; 52.4% had a medium-high economic condition, 33.4% a modest one and 14.2% some economic problems; 95% are married or cohabiting. Healthcare professionals and participants (mothers and fathers) took part in the study voluntarily and without compensation. The recruited subjects were given an Information Note with a description of the aims of the study and those who agreed to participate were asked to sign an Informed Consent Form. To protect their privacy, a code was assigned to participants, with which they became part of the study (they could authorize the communication of their names to the research centers). The research followed APA Ethical Principles of Psychologists and Code of Conduct. Participants were explained in detail all the objectives of the research and the methodology of analysis used. The study was approved by the Ethics Committee for Experimentation of the University of Padua (n. 53FB052AA456203CE7F4E9C76EBAFAEE).

## Methods and instruments

A mixed-methods design was adopted through the use of a self-report protocol and the implementation of an in-depth interview (Cena et al., 2022).

### The quantitative protocol

*A sociodemographic form* was used to collect age, nationality, education, profession, marital status, pregnancy data (gestational week, referral facility, number of pregnancies, any previous abortions), and perceived level of support from family, friends, and healthcare services.

*Prolonged Grief-13* (PG-13) (Prigerson et al., 2008) comprised of 13 items (2 dichotomous, 11 assessed on a 5-point Likert scale) was used to investigate the presence of prolonged grief symptoms. The result is calculated on the basis of five criteria: the event of loss; separation anxiety; the duration criterion; cognitive, emotional, and behavioral symptoms; and significant functional impairment after 6 months. A prolonged grief diagnosis is given when all five criteria are met. However, the instrument can also serve as a continuous measure by summing up the symptom items and excluding the two items concerning duration and impairment of functioning (Prigerson et al., 2008).

The analysis of internal consistency confirms that the single factor structure is highly satisfactory, with Cronbach’s α coefficient equal to 0.93. Therefore, the extraction of the only factor and good reliability analysis leads to the conclusion that the PG-13 can be considered a valuable instrument for the evaluation of the PGD in clinical practice (De Luca et al., 2015). In current study Cronbach’s alpha of PG-13 is 0.83 for female participants and 0.68 for male participants.

*The Parental Assessment of Paternal Affectivity* (PAPA) (Baldoni and Giannotti, 2020) uses a 10-point Likert scale to investigate paternal affectivity and the corresponding version for mothers. *The Parental Assessment of Maternal Affectivity* (PAMA) for mothers consisted of 10 items that investigated 8 dimensions: anxiety, depression, perceived stress, irritability/rage, relational problems (in a couple, with friends and at work), alterations in illness behavior (somatizations, functional medical disorders, hypochondriac complaints), physiological disorders (sleep, appetite, or sexual desire-related disorders), addiction disorders, and behavioral acts. The instrument thus makes it possible to identify fathers/mothers’ perinatal affective disorders, respectively.

Preliminary findings concerning the prenatal period showed significant association between PAPA total scores (*P* = 0.05) and single scale scores with many scores on CES-D, SCL-90-R, ASA, PSS, and DAS. Preliminary data of this Italian validation study confirm the PAPA as a useful tool for the screening of paternal affective disorders in the perinatal period (Baldoni et al., 2018). In the study described here, Cronbach’s alpha is 0.68 for PAMA and 0.66 for PAPA.

*The Dyadic Adjustment Scale short version* (DAS-4) (Sabourin et al., 2005b) was used to assess couple satisfaction. It consists of four items, three of which are on a 6-point Likert scale ranging from 0 (all the time) to 5 (never), while the final item is on a 7-point scale, ranging from 0 (extremely unhappy) to 6 (perfect).

The DAS-4 proved to be informative at all levels of couple satisfaction. Compared with the 32-item version of the DAS (DAS-32), it was as effective in predicting couple dissolution and was significantly less contaminated by socially desirable responding (Sabourin et al., 2005a). In the present research, the Cronbach’s alpha of the DAS-4 corresponds to 0.61 for female participants and 0.62 for male participants.

*The Daily Spiritual Experiences Scale* (DSES) (Currier et al., 2012) (16 items on a 6-point Likert scale) to examine an individual’s perception of transcendence and their interaction with it in everyday life.

The DSES evidenced good reliability across several studies with internal consistency estimates in the 0.90 s (Underwood and Teresi, 2002). In this study, the Cronbach’s alpha of the DSES for women is 0.93, while it is 0.90 for men.

*The Impact of Event Scale-Revised* (IES-R) (Weiss and Marmar, 1997) (22 items on a 5-point Likert scale) was used to measure the subjective response to the pandemic impact, assessing the presence and extent of post-traumatic stress disorder symptoms: intrusiveness, avoidance, and hyperarousal. The IES-R is a revised version of the IES and was developed because the original version did not include a hyper-arousal subscale. Both versions have shown good psychometric properties. Test–retest reliability (*r* = 0.89–0.94) and internal consistency (Cronbach’s α) for each subscale (intrusion = 0.87–0.94, avoidance = 0.84–0.97, hyper-arousal = 0.79–0.91) are acceptable. Correlations have been found to be high between those of the IES-R and the original IES for the intrusion (*r* = 0.86) and avoidance (*r* = 0.66) subscales, which supports the concurrent validity of both measures (Craparo et al., 2013). The Cronbach’s alpha of the IES-R in this study is 0.88 for female participants and 0.81 for male participants.

Self-report questionnaires were uploaded on an online platform. Data analysis was carried out using SPSS software.

### Qualitative analysis

The data collection was carried out through in-depth interviews, which allowed the researchers to understand the participants’ points of view on the investigated topic and draw on narratives about their experiences, attitudes, and perceptions. The interviews, lasting about 60 min each, were conducted *via* the Internet, recorded, and transcribed. The semi-structured interview investigated the following issues: history of pregnancy, communication of the loss, experiences related to grief, rituals, changes in the relationship with the partner, support received, the psychological impact of COVID-19, and the role of spirituality in the management of grief.

As required by the thematic analysis (Braun and Clarke, 2012), three phases were conducted. First phase: repeated listening to the audio recordings of the individual interviews and reading of their transcription to retrieve information about participants’ experiences, leading to the recognition of the main themes of their narratives. Second phase: interpretation of the meanings of the main themes and their links with the whole discourse and recognition of the perspectives of each individual participant, as well as the similarities and differences between them. Third phase: definition of the thematic dimensions (Testoni et al., 2019).

Thematic analysis is very flexible and is suitable for studying participants’ experiences and perceptions of a given phenomenon (Braun and Clarke, 2012) through an inductive process of coding and creating themes from the data without referring to any previous theory (Boyatzis, 1998).

Analyses were performed by a trained researcher under the supervision of an experienced qualitative analysis researcher. This made it possible to have two coders (inter-rater reliability) who clearly defined coding rules in advance, thus enabling the creation of a shared analysis codebook. There was total agreement between the two researchers. After they reached agreement on the interpretation, another researcher discussed the procedure and the results obtained. Finally, after modifications were agreed upon by the three researchers, the final structure of the report was defined.

The paper-and-pencil analysis operations were then integrated using the computer program qualitative analysis software Atlas.ti (Muhr, 1991) which has been precisely designed to aid researchers in qualitative data interpretation, allowing an analysis that is as objective as possible.

## Results

### Quantitative analysis

With respect to participants, there were four couples: Isabella–Antonio, Cristina–Ernesto, Laura–Giulio, and Valeria–Pietro. Five participants did not attend the interview (Barbara, Claudia, Elisa, Silvia, and Teresa).

### Results for female participants

With respect to the sociodemographic variables, the average gestation period was about 19 weeks (*M* = 18.7, *SD* = 12.4, Range = 2–42), the average number of previous pregnancies is one (*M* = 1.4, *SD* = 1.5, Range = 0–5), 43% had previous experience of miscarriage and for almost half of participants it was the first child. One-third of the group did not perceive sufficient support from family members, their network of friends, and especially the healthcare service. Half of the mothers stated that they received good support from their partners, while half defined it as absent or scarce.

IES-R: Seven participants (44%) present symptoms usually similar to post-traumatic stress disorder.

PG-13: All participants met criterion A, as all had experienced bereavement; 31.3% met criterion B, relating to separation distress, while criterion C, relating to the duration of bereavement symptoms, was met by 43.8%. Criterion D, relating to the presence of cognitive, emotional, and behavioral symptoms, was met by only 6.3%, and criterion E, relating to significant functional impairment 6 months after loss, was met by 31.3% of participants. However, none of the participants met all five criteria necessary for establishing the diagnosis, so none of the mothers showed complicated grief.

The PAMA: Those who had a score above 40 (Barbara = 40, Elisa = 56, Flavia = 60, Laura = 50) were at risk of an affective parental disorder.

The DSES: 50% of the participants had a high perception of transcendence, 19% had a slight perception, and the remaining 31% had little or no perception.

The DAS-4: 3 participants (19%) had a score below 13 and suffered from couple dissatisfaction.

### Results for male participants

The sociodemographic form showed that, for one, this was the first experience of pregnancy. Regarding the analysis of social support, for four of the fathers, the support received from family was good; in relation to the friendship network, three described it as good, while two described it as sufficient. Concerning the health services, two felt that the support was absent, one described it as poor, one as sufficient, and one as very good. Four noted the lack of psychologists and assistants. Four fathers pointed out that they could not access hospitals or have contact with doctors and paramedics. The lack of support was blamed on the COVID-19 restrictions.

IES-R: Three (60%) present symptoms usually similar to post-traumatic stress disorder.

PG-13: All participants met criterion A because they had suffered from grief. None met criterion B relating to separation anxiety. Criterion C, the duration of symptoms related to grief, was met by one. None met criterion D, relating to the presence of cognitive, emotional, and behavioral symptoms. None met criterion E, relating to a significant functional impairment 6 months after the loss. Thus, none of the participants fulfilled all five criteria necessary for the diagnosis, and none of the fathers showed a significant picture of prolonged bereavement.

PAPA: All participants scored below 40; thus, none of the participants were at risk of developing an affective parental disorder.

DSES: Two of the participants reported a slight perception of transcendence, while another two described it as moderate; one reported a high perception.

DAS-4: No participants scored below the clinical cut-off, indicating the absence of clinical distress related to their relationship with their partner.

### Results of correlations

Correlation analysis between all distress measures and between distress measures and other variables—maternal/paternal affectivity, couple satisfaction, spirituality, socio-demographic variables, pregnancy variables, and perceived support variables -, were performed using the Pearson r coefficient. All distress measures are significantly correlated: the PG-13-total show high positive correlation with the subscales of the IES-R avoidance (*r* = 0.52), intrusiveness (*r* = 0.57), hyperarousal (*r* = 0.55), and with the total (*r* = 0.68). The PAMA/PAPA total correlates positively with the PG-13 total score (*r* = 0.55) and IES-R intrusiveness (*r* = 0.44). The DAS-4 total correlates negatively with IES-R total (*r* = −0.50) and IES-R avoidance (*r* = −0.46). The DSES total positively correlates with IES-R avoidance (rho = 0.64). Considering socio-demographic variables, high education and high level of economic conditions correlate negatively with IES-R avoidance (*r* = −0.54 and *r* = −0.44, respectively) while the presence of previous children correlates positively with IES-R hyperarousal (*r* = 0.53) and with IES-R total (*r* = 0.46). Furthermore, considering the pregnancy data, gestational week correlates positively with IES-R hyperarousal (*r* = 0.71) and with IES-R total (*r* = 0.45). Finally, considering perceived support, high support from family and high support from friends correlate negatively with IES-R avoidance (*r* = −0.53 and *r* = −0.44, respectively). Spirituality was significantly correlated only to IES-R Avoidance (*r* = 0.64) (Table 1).

**TABLE 1:** Correlations of distress measures with maternal/paternal affectivity, couple satisfaction, spirituality and with socio-demographic, pregnancy, and perceived support variables (*N* = 21).

	Distress measures				
	Avoidance	Intrusiveness	Hyperarousal	IES-R total	PG-13 total
**Distress measures**					
Avoidance	−−				
Intrusiveness	0.45*	−−			
Hyperarousal	0.46*	0.55**	−−		
IES-R_Total	0.77***	0.87***	0.79***	−−	
PG-13 Total	0.52*	0.57**	0.55**	0.68***	−−
**Maternal/Paternal affectivity**					
PAMA/PAPA Total	0.38	0.44*	0.04	0.38	0.55**
**Couple satisfaction**					
DAS-4 total	−0.46*	−0.43	−0.31	−0.50*	−0.43
**Spirituality**					
DSES total	0.64**	0.08	0.24	0.37	0.39
**Socio-demographic variables**					
Gender (Female = 1; Male = 0)	0.08	0.01	0.02	0.04	0.29
Age	−0.21	−0.06	−0.09	−0.14	−0.11
Education (university degree = 1; lower level = 0)	−0.54*	−0.04	−0.21	−0.30	−0.13
Economic condition (medium-high level = 1; lower level = 0)	−0.44*	−0.01	−0.31	−0.28	−0.28
Previous children (Yes = 1; No = 0)	0.36	0.29	0.53*	0.46*	0.39
**Pregnancy variables**					
Gestational week	0.17	0.28	0.71***	0.45*	0.43
Number of pregnancies	0.11	0.04	0.40	0.19	0.40
Previous miscarriages/stillbirth (Yes = 1; No = 0)	−0.25	0.11	0.29	0.05	0.24
**Precived support variables**					
Support from family	−0.53*	−0.24	−0.26	−0.41	−0.31
Support from friends	−0.44*	−0.08	−0.41	−0.35	−0.40
Support from health services	−0.29	−0.04	−0.05	−0.15	0.10

*p < 0.05; **p < 0.01; ***p < 0.001.

### Results of participant’s gender differences

Gender differences on all variables—distress measures, maternal/paternal affectivity, couple satisfaction, spirituality, socio-demographic variables, pregnancy variables, and perceived support variables—were analyzed using the test t for independent samples. No gender differences were found except for age of participants (*t* = 2.44, df = 19, *p* = 0.012) and support of health services (*t* = −2.31, df = 19, *p* = 0.016). Male participants are older than female participants (*M* = 40.2, *SD* = 3.4 and *M* = 36.3, *SD* = 3.1, respectively), while male participants perceived less support of health services than female participants (*M* = 2.4, *SD* = 1.7 and *M* = 3.9, *SD* = 1.2, respectively).

Gender differences on correlations were analyzed by calculating the r-to-z transformation to compare correlations across men and women. No gender differences were found except for education with IES-R avoidance correlation (*z* = −2.53, *p* = 0.006) and previous children with PG-13 total (*z* = 2.45, *p* = 0.007). Male participants showed highest negative correlation between education and IES-R avoidance than female participants (*r* = −0.98 and *r* = −0.36, respectively), while they showed highest positive correlation between previous children with PG-13 total (*r* = 0.98 and *r* = 0.41, respectively).

### Qualitative analysis

Four areas of thematic prevalence emerged from the analysis of the interviews:

– Psychological Complexity of Bereavement: where the main grief-related experiences described by the participants were reported.
– The Impact of COVID-19: the thematic area refers to the difficulties experienced by parents related to the concomitance of the Covid-19 pandemic with loss.
– Disenfranchisement vs. Support: refers to the presence or absence of external support.

Relationship with Spirituality and contact with the lost child: the area concerns the role played by spirituality/religion in the elaboration of grief.

All the names cited are pseudonyms. In the brackets, only the significant results are reported the first time each name is quoted (Table 2).

**TABLE 2:** Thematic areas and sub-areas emerging from the qualitative analysis.

	First theme	Second theme	Third theme	Fourth theme

	Psychological complexity of bereavement	The impact of COVID-19	Disenfranchisement vs. support	Relationship with spirituality and contact with the lost child
First subarea	Bereavement-related experiences	The loneliness experienced by both partners due to restrictions	Disenfranchisement	Role of spirituality/religion in grieving
Second subarea	Confusion related to one’s parental identity	The difficulty in accessing services	Social support	Contact with the fetus and funeral rites

### Psychological complexity of bereavement

The first theme refers to grieving. The most frequent feelings were sadness, fear, anger, envy, guilt, and the sudden dissolution of expectations about the imminent construction of a family. This area was composed of two subareas.

The first subarea was inherent to the grieving, which involved both mothers and fathers in different ways. The body was the first dimension denouncing the loss. Sofia (IES-R = 34; DSES = 33) stated: “I have hallucinated pains, and this and this prevents me from returning to normal. My body and I are still quarreling.” The sense of an empty and arid womb was particularly painful, as described by Roberta (DSES = 40): “After the curettage, what you feel is a sense of emptiness, because you perceive that your body was changing, your abdomen swelled, and then a great sense of emptiness overwhelms you.” According to her, “My husband was worried, first of all, whether I would recover, and that’s why he didn’t want to grieve, hoping to support me.” Marianna said: “The body can resist physical pain, what is unbearable is the internal suffering. Something breaks inside me.” Valeria (IES-R = 49; DSES = 31), who also had a previous miscarriage/stillbirth, and the new baby was born dead after 9 months due to dystocia, narrated: “I was petrified, I couldn’t say anything, think about anything, or cry. Even today, at times, I deny what happened. “She tried to understand her partner Pietro”: “He’s a racer, so he’s raced a lot, worked a lot, outlined technical projects. I understood that this was his way to face the loss.” The participants also suffered from intense anger. Flavia (IES-R = 53; PAMA = 60; DAS-4 = 10), who had experienced a previous miscarriage, reported: “I’m absolutely angry because, very nicely, the gynecologist told me, ‘Well madam what do you want me to tell you, there is no longer the baby’s heartbeat.”’ Her relationship with her husband went into crisis: “Because of constant guilt and anxiety. In the end, I had to give him an ultimatum.” Miriana stated, “I am angry. If they had realized before that the fibroma was already big, what would have happened?” And Flavia said: “I am angry with karma and with God. Two very dear friends of mine found out they were pregnant, but they did not accept it. Seeing them not happy and me instead who would have strongly wanted it disturbs me profoundly.” Pietro, Valeria’s husband, similarly said: “I had never felt an emotion like this, I think the right name is anger; it was explosive at the beginning, I felt this energy, I was not willing to tolerate this loss’.” The common emotion between mothers and fathers was a sense of guilt. Additionally, fathers showed an intense feeling of guilt, as in the case of Ernesto (IES-R = 41; DSES = 29), who had to make a difficult choice when, with his wife Cristina, he discovered that the fetus had a severe malformation at the fifth month: “It was a terrible choice that I didn’t expect to make. I still live with this choice in my mind. I have regret, and the doubt persists. Did we get it right, or did we get it wrong?” Fathers also felt intense existential suffering, as in the case of Antonio (IES-R = 41), who affirmed that “I always ask me what I have lived, what happened, what it could have been. Now there is a feeling of sadness, of emptiness, of a physical lack, because I wanted another child.” However, the most important feeling was the sense of responsibility and a need to be useful to recovering normalcy, as affirmed by Alberto (IES-R = 41): “She prefers to talk about it to get over that moment, while my personal protection is to deflect the subject. […] I also had to take care of our son, and it wasn’t easy to explain to him what had happened”; and Pietro “My reaction is to do, to act, my wife on the other hand needs time, to stay calm, to put her thoughts in order, to try to accept this loss.”

The second sub-area was inherent to confusion related to one’s parental identity, especially among mothers who have had previous miscarriages or stillbirth. The participants were confused about their role, they did not know whether they should consider themselves mothers or not. Sofia, who had already previously had two similar experiences, said, “I wasn’t able to manage pregnancy. Ambivalence characterized my entire pregnancy; I was pregnant and I drank a glass of wine, I was pregnant and I started to smoke again, I was pregnant and I took a drug to sleep, always with the thought that I was doing damage, that I was not an adequate mother. Moreover, I didn’t want to see the baby during expulsion, and I said, ‘what kind of mother are you? Why don’t you want to see your child?’ So finally, I chose to see him.” She also said, “It is not the events themselves that upset me the most, but seeing becoming a mother fade away, which is becoming more and more impossible by now; all this gives strong frustration.” Similarly, Marianna, who had a previous miscarriage, affirmed, “I want a child, so I asked myself what the meaning of my life was. I thought there wasn’t one; I wasn’t working and, above all, I wasn’t a mother.” Gioia also had the same feeling: “For a while, I feel like a mother who has a child. On these days, I would wonder, if 1 day I will have children and they ask me how many children you have had, will I have the courage to count the miscarried ones? I’m really confused.” Cristina (DSES = 47) said: “I had the perception that I was not a mother because I had abandoned my child, both after the birth and after the burial.”

### The impact of COVID-19

The second thematic area was related to the concomitance of pandemic and miscarriage and stillbirth. It was divided into two subareas: the loneliness experienced by both partners due to restrictions and the difficulty in accessing services.

The first subarea considered the experiences of mothers and fathers, who could not be near their partner during visits, thus fueling a sense of loneliness and exclusion. The couple also did not have the opportunity to confront each other when making important decisions as the restrictions did not allow fathers to attend the various visits. Flavia, who had problems related to the infections, tells: “I was terrified of being pregnant, alternating with happiness at the news after the COVID nightmare, but given everything that was happening, I was terrified that something might happen.” The other participants reported that the main consequence of the pandemic was that they had to make their visits alone and be hospitalized and operated on in the absence of their partner or parents, as well as having to make important choices alone, as in the case of Antonio’s wife Isabella, who said, “The phone, the video call maybe, were not enough, and maybe the most fundamental part for me was the emergency room, when you really had to realize what was happening, and my husband was out there. It made me so sad.” Fathers also tell, who, on the other hand, suffered from an inability to attend the visits and participate in their partners’ hospitalizations, exacerbating their sense of helplessness, as described by Antonio: “I was just there waiting for my wife for 48 h, I did not have the ability to access the hospital. My biggest regret is not having seen my son and not having been with my wife in that terrible moment.”

The pandemic restriction also exacerbated the distress, as explained by Cristina: “It was difficult to go for a walk, it seemed to me that the house was closing in on me, as if the walls were collapsing and the pain was becoming even more vivid,” and by Roberta: “Without meeting people, I had too much time to think, and this had broken me down. I felt that my head would explode because I was suffocating.”

A huge sense of protection toward the partner derived from this situation, as described by Giulio (DSES = 40) with respect to his wife Laura: “Mainly I was worried, not so much for the loss of the child, but for what was happening to my wife. I was worried about her physical and psychological conditions,” and by Antonio: “A huge feeling of protection toward my wife took over, because I wanted her to feel well since the beginning, regardless of what could have happened with the pregnancy.”

The second subarea considered the difficulty in accessing services and medical examinations. Moreover, it was very difficult to access the necessary controls, as described by Marianna: “My gynecologist told me that she couldn’t receive any more patients for examinations. Trying to call the hospital, I received only negative answers. Everything was blocked; the answer I was given was to go to a private professional, and this upset me further.”

Furthermore, as stated by Ernesto: “There was a lack of concrete support: finding the doctors, going to the hospital. The exams were continuously postponed, extensive waiting times, calling and not finding anybody,” and by Alberto: “My wife was not assisted at all, and the operators did not even let me stay in the waiting room.”

### Disenfranchisement vs. support

This area is divided into two subareas: disenfranchisement and social support.

The first subarea concerns disenfranchisement, which caused great suffering. Participants did not always feel their grief and bereavement was acknowledged from the outside and sometimes received superficial or devaluing comments about the experience from acquaintances, friends and family members and even from their partners, a factor that exacerbated the pain of the moment. Marianna perceived her partner as absolutely absent and detached: “He didn’t suffer as much as I did, and he didn’t talk about it, because it was an uncomfortable subject. For him, this is not a problem.” In Flavia’s opinion, “People don’t give importance to this experience and think that I already have a daughter and so I must be satisfied! For this reason, I prefer to avoid people who know me.” Similarly, Marianna did not want to tell anyone about this loss: “I decided not to talk to anyone about my condition for fear of not being understood. However, I needed someone to ask me if I needed help.” According to Ernesto, “You can only talk about it if someone shows interest. This wound is very painful and the others don’t understand it, so they can’t help me.” Similarly, Pietro said, “Some people feel the need to ask questions, disrespecting you. Their language goes faster than their thoughts because they are curious. There is a terrible level of emotional ignorance and lack of empathy.” The medical staff also proved unable to recognize the mothers’ suffering, as Cristina continued: “The doctors don’t recognize your pain. They are cold, detached, even when they give you the bad news.”

The second subarea concerned the importance of support. If, on the one hand, participants did not always feel understood by others, receiving comments from outside aimed at minimizing the loss, on the other hand, they always found figures within their own circle of friends, family or care team who supported them. For many of them, social support was an important protective factor in the grieving process, as described by Isabella: “I was lucky because the nurse was very close to me and allowed me to go out to see my husband,” and Miriana: “I met a fantastic team. Everyone tried as much as possible to overcome the limitations of the pandemic to make me feel more comfortable. Their sense of humanity prevailed.” Those who were supported by mutual-aid groups had a great advantage, as Valeria emphasized: “Participating in these groups allowed us to find out that many other couples had the same experience, and we found great support in these people especially, because they understand.”

Most fathers and mothers, however, considered the support offered by family and friends as adequate and comforting.

### Relationship with spirituality and contact with the lost child

Religiosity/spirituality was not particularly effective in supporting the grief experience. Sofia (IES-R = 34; DSES = 33) stated, “I don’t have a religion to take refuge in. I think things are the way they are, and I deal with them the way they are. I consider myself unlucky and do not consider any existence of this baby.” In the same vein, the fathers also have a low level of spirituality. According to Giulio (DSES = 40), “‘It had to be like this,’ it is a scenario that is contemplated when you think about having a child, it is a natural thing that can happen.” Similarly, Ernesto (IES-R = 41; DSES = 29): “I don’t find support in religion and I don’t feel like a particularly unfortunate case and I’m not desperate. I feel very lucid because I believe that this is a human thing, that it can happen.” Both lower and high levels of spirituality are accompanied by a positive experience of contact with the fetus and the funeral ritual, as in the cases of Cristina (DSES = 47): “I’m so pleased, I wanted to see it because now it’s not nebulous; I have a concrete face to remember, which at the beginning occupied an immense space in my mind and is now slowly getting smaller, taking the right proportions,” and Valeria (IES-R = 49; DSES = 31): “I needed to touch, to see, to give a face, to give a weight, even holding her in my arms helped me. I would have lacked contact with reality if I had not seen her. […] In this way I closed the circle,” as well as Miriana: “Being able to touch and hold him allowed me to understand what happened,” and Flavia (DSES = 51), who also treasured the funeral: “In this way I was able to say goodbye to him, as we do to those who have lived with us.”

For the fathers, it was easier not to celebrate any funeral, as in the case of Ernesto (DSES = 29): “The funeral makes sense if you can remember who died, if you do it for someone you lived with. I knew he was going to be stillborn because we had chosen to have him stillborn because of his severe malformation, so it was better not to proceed further,” and Alberto (DSES = 58): “I suffered a lot for this loss. I preferred not to have any funeral because if I had to think about the burial, it would have been much harder.”

## Discussion

This study adopted a mixed-method research design. The quantitative analysis exhibited that a large number of participants presented most of the symptoms attributable to post-traumatic distress, moreover, several participants showed low levels of couple satisfaction and disturbances in the affective parental sphere. Correlational analysis revealed significant relationships between the presence of symptoms of post-traumatic stress disorder and the dimensions of hyperarousal and intrusiveness. Although all participants are suffering intently from the loss, none could be diagnosed as having complicated grief, and the total PG-13 score correlated with all the dimensions of the IES-R, showing that a higher level of distress corresponded to a greater difficulty in processing the loss. These results are in line with the literature emphasizing that miscarriage is an extremely traumatic experience (Farren et al., 2016). The results indicate that greater difficulties in grieving are positively correlated with emotional difficulties in the parenting role, having had children previously, and when gestational age was longer. With respect to gestational age, the results are in line with Cuisinier et al. (1993) but in contrast with some studies that underline that the suffering is the same in all the phases of pregnancy (Callister, 2006; Kersting and Wagner, 2012). This issue is less studied, and the limited results are inconsistent, so it would be useful to conduct further research.

From the qualitative analysis, four areas of thematic prevalence emerged, with some subareas. Psychological complexity of bereavement was the most important because participants preferred to describe their psychological experience. It was composed of two subareas: the grieving experience and the couple relationship. The second area, the impact of the COVID-19, was composed of two subareas: the experiences of mothers and of fathers. The third, disenfranchisement vs. support, considered the two experiences in two different subareas. Finally, spirituality is positively correlated with avoidance, and this result indicates the ambivalent role of this dimension, that did not seem to help parents in grieving. Indeed, this dimension did not signify the contact with the lost children and whether the relationship with transcendence influenced the relationships with them. With regard to grieving, it was confirmed that there were different ways of dealing with loss between mothers and fathers. The women considered it necessary to have time for introspection and reflection, and it seemed to help to have positive conversations. Conversely, the male tendency was to adopt elusive strategies, avoiding talking about it or engaging in other activities that allowed them not to focus on the problem (Obst et al., 2020). Specifically, while women described a great deal of grief, demonstrating the presence of all the stages of mourning described by the Kübler Ross model (Corr, 2019), as already described in the literature (Testoni et al., 2020), men preferred silence and did not share their grief with their partners (Miller et al., 2019), probably due to culturally established gender roles and stereotypes (Rinehart and Kiselica, 2010), which also limited them in seeking support in family and friendship networks (Saunders and Peerson, 2009). This imbalance may partly explain why the DAS-4 results reported that several participants suffered from relationship problems.

The second thematic area was divided into the negative impact of COVID-19 on both maternal and paternal bereavement experiences because of indirect effects related to the difficulties in accessing healthcare services and follow-up visits, the lack of adequate support from social and healthcare personnel, and the inability of fathers to assist and offer support at the time of their wives’ hospitalizations. None of the participants, in fact, felt that Covid-19 had a direct effect on their pregnancy, even in cases where the women were infected. The main consequence of the pandemic was found in the fact that mothers were forced to make their visits alone, as well as being hospitalized and operated on. In fact, all of this took place without the presence of their partners or parents: this context burdened their experience and exacerbated their sense of loneliness and abandonment. Moreover, in some cases, the women found themselves making important decisions alone, without being able to communicate except by telephone. Participants felt, precisely because of the COVID, that they had lost an important piece of their experience, namely sharing with their partner. Finally, they felt very frightened about not being able, in some cases, to undergo check-ups on time.

Finally, the presence of Covid also weighed on the grieving process; the restrictions due to the infection prevented many from going out, distracting themselves, resuming work and having contact with the outside world.

The results are in line with the literature (Burki, 2020; Roberton et al., 2020), which shows that the negative effects of the pandemic on perinatal health are not limited to morbidity or mortality caused directly by the virus, but also by the restrictive measures that undermined social relationships, and thus support, especially inhibiting access to health services. Indeed, the literature has already shown that the pandemic was very stressful for fathers (Baldoni and Giannotti, 2020) and especially for pregnant women, who reported higher levels of anxiety and depression compared to cohorts analyzed in the pre-pandemic period (Dennis et al., 2017; Woody et al., 2017; Cena et al., 2020, 2021b; Yan et al., 2020), partly due to the lack of support from the healthcare system (Thayer and Gildner, 2020). The negative effects of the pandemic on maternal and perinatal health include restrictive measures, disruption of health services, and fear of using these services, which are among the main factors that compromised the physical, psychological, and social wellbeing of participants, similar to those facing pregnancy during the height of the pandemic (Burki, 2020; Roberton et al., 2020). Linked to this is an additional factor: the altered family and social relationships due to estrangement from loved ones and friends (Khalil et al., 2020; Chmielewska et al., 2021). Social support is important, but most participants perceived the disenfranchisement of their grief, on the part of both family and friends, and especially by health professionals (Lang et al., 2011). Perinatal grief was, in fact, misunderstood because if there were already children in the family, people said that they could be enough and, in any case, that there remains the possibility of trying to have more. Unfortunately, these perceptions do not consider that, in the representation of the mother and sometimes also of the father, the fetus is already personified and considered a child. This phenomenon has also been found to be widespread in the literature: according to Lang et al. (2011), many couples report that most friends and relatives are not even aware of important dates, such as the birth of the child or the anniversary of the loss; furthermore, it appears that health professionals often adopt a depersonalizing attitude toward the couple and the child, which conveys a feeling of devaluation toward this type of loss. This lack of support contributes to exacerbating the feelings of isolation and mourning experienced by parents.

The interviews also reveal that men feel they have to silence their pain, as their role is to accept and listen to their partner’s suffering. This is in line with the literature according to which men perceive that they cannot share their pain with their partner (Miller et al., 2019); furthermore, the freedom to openly manifest this suffering is also influenced by the lack of recognition of the man’s loss, based on social expectations that men should be strong and impassive and have the sole responsibility to act as a support for their partner (Rinehart and Kiselica, 2010; Due et al., 2017). This also seems to be linked to less support-seeking among both the friendship and family network (Saunders and Peerson, 2009).

Despite spirituality/religiosity being widely considered very helpful in managing grief (Park and Halifax, 2011), in this group, this dimension did not result in support. All the mothers who did not participate in the interview (Barbara, Claudia, Elisa, Silvia, Teresa) and those who obtained higher scores in PAMA (Barbara, Elisa, Flavia, Laura) were characterized by the highest spirituality in parallel to high levels of posttraumatic symptoms and low couple satisfaction. This suggests that not only is the couple’s relationship important but also that there may be a spiritual conflict with respect to the loss of the child (cf. Testoni et al., 2021) and that all this could have made it very difficult to talk about the experience. The inauspicious and often traumatic outcome of pregnancy has often led to a break, albeit temporary, with one’s spiritual dimension, especially among those who have always entrusted their lives and destinies to the divine plan. On the other hand, it was highlighted by participants in the interviews that neither the religious nor spiritual dimension informed the desire to see or touch the miscarried or stillborn fetus or mourn it through a funeral rite. Indeed, the literature shows that religiosity helps to overcome distress through the social support of the religious community, where grievers can share languages and symbolism to process the sense of loss (McIntosh et al., 1993; Arshad and Hafeez, 2016). This is in line with the study by McIntosh et al. (1993), according to which religious communities can be a supportive resource, just as greater religious participation seems to be correlated with a greater perception of social support, helping to decrease distress related to parental bereavement. It is possible that the restrictions imposed by the pandemic reduced the positive effect of this aspect.

## Conclusion

The results of the study show how the pandemic negatively impacted couples’ experience of miscarriage due to indirect consequences related to difficulties in the healthcare environment and restrictions imposed to prevent contagion. The restrictions exacerbated the negative effects of the trauma by making relationships with healthcare professionals more difficult and limiting contact with friends and family. In addition, decreased contact with participants’ religious communities diminished the power of that social support. The need for support was not compensated for by adequate psychological services either. It is imperative to equip gynecology and obstetrics departments with at least one staff psychologist because this professional could have provided the necessary help, even in the most difficult moments of the pandemic.

The most important limitation of this study is the fact that the majority of fathers did not want to participate, so it is possible that the results obtained by those who did participate are not indicative of a specific male method for dealing with this loss. In addition, The group of participants was small due to the fact that some parents who were contacted by the healthcare professionals did not agree to participate in the study because the perinatal loss event was traumatic and painful and they did not want to talk about it again. Therefore, the small number of participants did not allow for more in-depth statistical analysis, and results cannot be generalized. Furthermore, most of the sample is Italian, has a university degree, and medium-high economic conditions. Then, results are not representative of a larger population.

Additional studies could further survey the relationship between grieving and gestational age and with religiosity/spirituality.

The present study has as its future objective the administration of the instruments at a distance of 6 months: it would be useful to carry out a follow-up with the aim of verifying the occurrence of significant changes, also in relation to the evolution of the pandemic context. Furthermore, another future objective is to extend the research to a larger number of participants, who have also suffered perinatal losses beyond the time period considered so far; connected with this objective, extending the number of participants could make it possible to recruit a substantial number of couples, which would allow an analysis not only of individuals, but also between partners.

The participants also pointed out that social support was crucial for them in the process of processing the loss but that in some circumstances the social network was not able to recognize the underlying suffering of the loss m Considering mothers’ need to express their grief and to receive strong support, together with the need to mentally and psychologically reorganize the way they represent their deceased child, further research should focus on what professional interventions could be particularly helpful in supporting the grieving process, especially by social workers who can improve social support. Finally, future research should investigate the reactions of siblings and fathers to perinatal loss, since this type of bereavement is still poorly recognized by society, in order to develop a more appropriate support system for them as well.

## Data availability statement

The raw data supporting the conclusions of this article will be made available by the authors, without undue reservation.

## Ethics statement

The studies involving human participants were reviewed and approved by the Ethics Committee for Experimentation of the University of Padua. The patients/participants provided their written informed consent to participate in this study.

## Author contributions

IT and LC: conceptualization, methodology, writing—review and editing, and supervision. EI and AT: software. IT, LC, AT, GL, LR, and EI: analysis. IT, LC, GL, EI, and AT: investigation. GL: resources. IT, LC, EI, GL, AT, and LR: data curation. IT, LC, GL, EI, AT, NT, and LN: writing—original draft preparation. IT, LC, EI, and AT: project administration. All authors have read and agreed to the published version of the manuscript.
